# Coronavirus Usurps the Autophagy-Lysosome Pathway and Induces Membranes Rearrangement for Infection and Pathogenesis

**DOI:** 10.3389/fmicb.2022.846543

**Published:** 2022-03-02

**Authors:** Haowei Liang, Dan Luo, Hai Liao, Shun Li

**Affiliations:** ^1^Department of Immunology, School of Basic Medical Sciences, Chengdu Medical College, Chengdu, China; ^2^School of Life Sciences and Engineering, Southwest Jiaotong University, Chengdu, China; ^3^Non-coding RNA and Drug Discovery Key Laboratory of Sichuan Province, Chengdu Medical College, Chengdu, China

**Keywords:** coronavirus, double membrane vesicles (DMV), membranes rearrangement, virus escape, autophagy-lysosome pathway

## Abstract

Autophagy is a crucial and conserved homeostatic mechanism for early defense against viral infections. Recent studies indicate that coronaviruses (CoVs) have evolved various strategies to evade the autophagy–lysosome pathway. In this minireview, we describe the source of double-membrane vesicles during CoV infection, which creates a microenvironment that promotes viral RNA replication and virion synthesis and protects the viral genome from detection by the host. Firstly, CoVs hijack autophagy initiation through non-structural proteins and open-reading frames, leading to the use of non-nucleated phagophores and omegasomes for autophagy-derived double-membrane vesicles. Contrastingly, membrane rearrangement by hijacking ER-associated degradation machinery to form ER-derived double-membrane vesicles independent from the typical autophagy process is another important routine for the production of double-membrane vesicles. Furthermore, we summarize the molecular mechanisms by which CoV non-structural proteins and open-reading frames are used to intercept autophagic flux and thereby evade host clearance and innate immunity. A comprehensive understanding of the above mechanisms may contribute to developing novel therapies and clinical drugs against coronavirus disease 2019 (COVID-19) in the future.

## Introduction

At the beginning of the 21st century, we have witnessed the outbreak of three types of diseases by coronavirus (CoV) infection, which are severe acute respiratory syndrome (SARS), Middle East respiratory syndrome (MERS), and coronavirus disease 2019 (COVID-19). CoVs have caused serious public health problems and economic losses. Although various countries have begun to vaccinate against SARS-CoV-2, there is still an urgent need to find effective antiviral drugs to treat COVID-19 (de Wit et al., 2020). Therefore, it is crucial to discover new drug targets according to viral replication and infection in host cells.

Coronaviruses (CoVs) are enveloped viruses with a positive-sense, single-stranded RNA (ssRNA) genome. Although the specific process of CoV infection of host cells is not yet completely clear, according to previous studies, the infection cycle of CoVs is divided into several steps: attachment and membrane fusion, uncoating of the viral genome, translation of open-reading frames (ORFs) and non-structural proteins (NSPs), formation of double-membrane vesicles (DMVs), genome transcription and replication, and virion release (Fung and Liu, 2019). Once the virus enters the host, the two polyprotein precursors, pp1a and pp1ab, are directly translated (Heer et al., 2020). Then, at least 16 NSPs are produced due to the serial cleavage of pp1a and pp1ab (Hufsky et al., 2021). NSPs generally are localized at the endoplasmic reticulum (ER) to establish the DMVs for viral replication (Benvenuto et al., 2020). DMVs located in the perinuclear region of the host cells provide a microenvironment that promotes viral genome replication and protects the virion from clearance (Gui et al., 2019).

Autophagy is a conserved cellular process for maintaining cell homeostasis and eliminating misfolded proteins, damaged organelles, and pathogens (Yue et al., 2018). The autophagy signaling pathway consists of three steps: the formation of the initiation complex, the development of mature autophagosomes, and fusion with the lysosome (Fung and Liu, 2019; Ghosh et al., 2020). Viruses could lead to the generation of an autophagic response to viral infection. For most viruses, knocking out the autophagy-related genes (ATGs) would increase viral titers (Ismayil et al., 2020; Lal et al., 2020). Generally, double-stranded DNA (dsRNA) viruses activate the tripartite motif protein 32 (TRIM32)/tax1-binding protein 1 complex through TIR domain-containing adapter molecule 1 (TRIF). Then, TRIF induces the autophagy–lysosome pathway to digest the virus by inhibiting Bcl-2 (Zhang et al., 2019). However, CoVs target different steps of the autophagy–lysosome pathway during infection (Zhao et al., 2021). Here, we suggest two main purposes for CoV autophagy evasion: the formation of protective structure DMVs and the suppression of autophagy flux by blocking the fusion of the autophagosome and the lysosome.

This minireview first summarizes the generation of DMVs, particularly the mechanism of CoV-induced rearrangement of the cellular membrane. Next, we suggest the role of NSPs and ORFs in suppressing autophagy–lysosome fusion and cargo degradation. This minireview provides a comprehensive understanding of the mechanisms by which CoVs evade the autophagy–lysosome pathway. We hope it can contribute to discovering new drug targets and therapeutic strategies for COVID-19 in the future.

## Coronaviruses Hijack Autophagy Initiation and ERAD Machinery to Form Double-Membrane Vesicles by Inducing Membrane Rearrangement

The autophagy–lysosome pathway serves as the first line of defense against viral genome replication and expression but also as a potential source of host membrane (Vabret et al., 2020). The production of CoV particles is positively correlated with the number of DMVs (Maity and Saha, 2021; Miao et al., 2021). In recent years, the formation and development of DMVs have attracted enormous attention due to the similarities between DMVs and autophagosomes. However, the relationship between DMVs and the autophagy pathway has been controversial. Recent studies have revealed that inhibition of autophagy blocks viral replication, which implies that the autophagic structure is required for viral replication (Olzmann and Carvalho, 2019). However, according to accumulated studies, the generation of DMVs does not depend on autophagy. Based on existing reports, we suggest that autophagy-derived DMVs and ER-derived DMVs are two different important pathways of DMV production. On the one hand, CoVs can hijack autophagy initiation to form unique DMVs. On the other hand, CoVs induce ER-derived DMVs independent of the autophagic pathway by hijacking host cell ER-associated degradation (ERAD) machinery (Konno et al., 2020).

According to recent reports, the NSP3 and NSP6 proteins of SARS-CoV-2 and MERS-CoV induce the formation of omegasome intermediates resident to the ER to form autophagy-specific phagophores, which results in the activation of the autophagy–lysosome pathway (Mihelc et al., 2021). Moreover, N protein and ORF8 of all three CoVs can activate the ULK1 complex to promote omegasome formation *via* the AMPK/mTOR signaling pathway (Snijder et al., 2020). SARS-CoV ORF8b can enhance autophagy initiation by inducing the ER stress signaling pathway (Yang and Shen, 2020). DMVs mimic autophagosomes in structure. Non-lipidated LC3-I is associated with the outer membrane of the DMVs. However, the autophagosome-like DMVs do not contain LC3-II and lose the ability to fuse with lysosomes (Liu et al., 2019; Vera-Otaroola et al., 2020). It has been reported that ORF3a of SARS-CoV-2 hijacks the lipidation of LC3, which inhibits the phosphatidylethanolamine (PE)-dependent conversion of LC3-I to LC3-ll (Zou et al., 2019). The reduction of LC3-ll leads to the inhibition of the conversion of nucleating phagophores to autophagosomes. Additionally, NSP6 and the PLP2 domain of NSP3 of SARS-CoV and MERS-CoV promote the degradation of Beclin1 through the activation of SKP2 *via* the AKT signaling pathway, which inhibits vesicle nucleation and autophagosome formation (Gassen et al., 2019). Therefore, these non-nucleation phagophores and omegasomes self-use to autophagy-derived DMVs. CoVs can utilize autophagy-derived DMVs to protect their DNA and proteins from being cleared by lysosomes, which provides a venue for CoV replication.

Typical autophagosomes originate from the ER-Golgi intermediate complex (ERGIC) and consist of various ER/Golgi-maker proteins, such as EDEM1, COP-ll, and RPN1. Therefore, the ER-Golgi is another important way to produce DMVs independent of the autophagy pathway. There is substantial evidence that CoVs induce membrane rearrangement by hijacking the host cell ERAD machinery (Miller et al., 2020; Qu et al., 2021). NSPs regulate ER and Golgi apparatus rearrangement to form DMVs directly from the ER (Gassen et al., 2019). CoV infection induces the accumulation of OS-9, EDEM1, and ERAD in the DMVs (Li et al., 2019). SARS-CoV-2 NSP4 and NSP6 induce ER-derived DMVs independent of the autophagic pathway by hijacking the host cell ERAD machinery (Muscolino et al., 2020). In addition, prior studies indicate that SARS-CoV NSP2 and MERS-CoV NSP3 could induce extensive rearrangement of cellular autophagosome-like vesicles around the ER and the Golgi apparatus (Pradel et al., 2020). The majority of host cells undergo ER stress after CoV infection. ER stress may be another target of NSPs, and it may be used as a driving force to promote the generation of DMVs and autophagosomes (Fung and Liu, 2019; Jin et al., 2020). SARS-CoV-2 ORF3-a has been reported to associate with non-lipidated LC3-I by activating ER stress, which promotes multiple-lamellar membrane structure emergence from the ER membrane, and prevents the generation of single-membrane structures (Alsaadi et al., 2020). Moreover, NSP6,7 of SARS-CoV-2 and NSP5,6 of SARS-CoV have been reported to block the EDEMsomes fusion with the late endosomes and then induce the membrane rearrangement to produce ER-derived DMVs (Maity and Saha, 2021; Tiwari et al., 2021). Therefore, membrane rearrangement by hijacking ERAD machinery to form ER-derived DMVs independently is another important way for viral replication.

**FIGURE 1:**
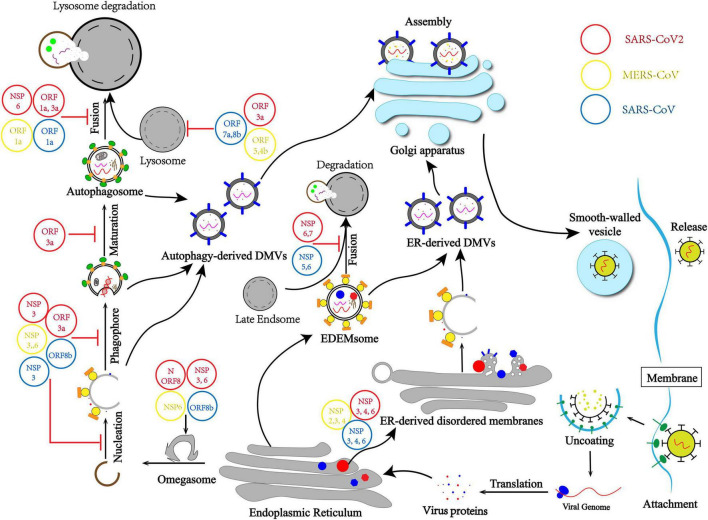
Coronavirus (CoV) usurps the autophagy–lysosome pathway and induces membrane rearrangement for infection and pathogenesis. CoVs hijack the initiation differently, leading to non-nucleation phagophores and omegasomes self-use to autophagy-derived double-membrane vesicles (DMVs). CoVs usurp lysosome degradation by modulating lysosomal acidity or preventing autophagosome–lysosome fusion. Moreover, membrane rearrangement by hijacking ERAD machinery and blocking the EDEMsome and late Endsome fusion to form endoplasmic reticulum (ER)-derived DMVs independent from the autophagy process.

Here we draw a mechanism model that describes how CoVs promote the generation of DMVs by inducing membrane rearrangement by different routines. First, CoVs hijack autophagy initiation and usurp the autophagy–lysosome pathway to form autophagy-derived DMVs. Second, CoVs induce ER-derived DMVs independently by hijacking ERAD machinery (Figure 1).

## Coronaviruses Usurp Host Clearance by Intercepting Autophagy Flux

The autophagy–lysosome pathway is a part of innate immunity, the first-line defense for eliminating pathogens. As part of the cellular defense system, autophagy protects against bacterial or viral infection by forming autophagosomes that encapsulate pathogens and transporting these cargoes to lysosomes for degradation (Zhao et al., 2021). Although CoVs hijack different steps of autophagy to promote DMV formation, there are still many autophagosomes produced in which viral RNA and proteins are enveloped and sent to the lysosomes for clearance (Vabret et al., 2020). Latest reports indicate that when autophagy initiation was induced during CoV infection, the autophagy flux surprisingly was blocked (Krafcikova et al., 2020; Miao et al., 2021). Studies report that failure of lysosomal fusion with autophagosomes has been observed. Therefore, CoVs can escape the clearance of the autophagy–lysosome pathway by intercepting autophagy, a crucial component of CoV replication and survival.

A recent study shows that the expression of lysosomal membrane genes and lysosomal acidification genes is downregulated in Vero cells infected with CoVs (Gassen et al., 2019). Moreover, lysosomal enzymes are inactivated, and changes in lysosomal acidity have been observed in CoV-infected Vero cells (Ghosh et al., 2020). Further studies were performed to elucidate the underlying molecular mechanism. According to a recent study, ORF7a and ORF8b could directly regulate the pH of lysosomes in SARS-CoV–infected Hela cells (Hayn et al., 2021). ORF5 and ORF4b of MERS-CoV could downregulate the expression of the lysosomal membrane and lysosomal acidification genes (Singh et al., 2021). Previous studies suggested that SARS-CoV-2 NSP6 and ORF1a in all three CoVs could reduce the autophagosome–lysosome fusion (Hayn et al., 2021). Moreover, recent studies reveal the mechanism by which SARS-CoV-2 ORF3a blocks autophagosome maturation and the fusion of the autophagosome with lysosome by inhibiting the assembly of VAMP8 (a significant vesicle adaptor protein) complex through the regulation of a membrane fusion accelerator (such as HOPS, VPS39, and STX17) (Miao et al., 2021). In addition, MERS-CoV NSP6 can regulate Beclin1 ubiquitination, an important protein for autophagosome–lysosome fusion (Wang et al., 2020). Moreover, the PLP-TM domain of NSP3 in all three CoVs binds Beclin1 and STING1 (stimulator of interferon response cGAMP interactor 1), which prevents Beclin1 from promoting autophagosome–lysosome fusion and inhibits the production of interferon (Zhang et al., 2021). Autophagy plays a pivotal role in IFN-I production (Zhu and Zheng, 2020). The NSP3 PLP-TM domain is necessary to complex Beclin1 to STING1, which prevents the stimulation of IFN production (Zhang et al., 2021). In the absence of IFNs, it is difficult for host cells to eliminate the virus through innate immunity (Pradel et al., 2020).

Therefore, there are two main purposes of CoVs intercepting autophagy flux. Firstly, CoVs usurp lysosome degradation by modulating lysosomal acidity or preventing autophagosome and lysosome fusion, which means CoVs evade the host first-line defense and protect viral DNA and proteins from being cleared (Figure 1). Secondly, the autophagy–lysosome pathway plays a pivotal role in IFN-I production as part of innate immunity. Blocking the autophagy flux is also important for CoVs usurping innate immunity elimination.

## Discussion

As a ssRNA virus, CoVs have spread globally, posing a serious threat to global public health. Major CoVs include SARS-CoV, MERS-CoV, and SARS-CoV-2. In most cases, CoVs possess the ability to usurp host clearance (Fung and Liu, 2019). Therefore, it is urgent and important to study the regulatory mechanism of virus evasion. Although CoVs evade the autophagy–lysosome pathway and innate immunity has been reported, many molecular mechanisms remain unclear, and many studies remain controversial (Pan et al., 2020). Many investigations have been conducted to determine the origin of DMVs and if autophagosomes are required for viral replication. This minireview aimed to elaborate on the relationship between DMVs and autophagosomes and the relationship between viral replication and autophagy. We also summarized the source of DMVs and how CoVs evade the autophagy–lysosome pathway (Figure 1).

Double-membrane vesicles protect virial DNA and proteins from being eliminated by lysosomes. The production of virus particles is positively correlated with the number of DMVs. Previously, there has been no distinction between autophagosomes and DMVs. Although DMVs and autophagosomes are similar in structure and co-localize with autophagy-related proteins, they differ in size, structure, and function (Nguyen et al., 2020). In this review, we summarize the source of DMVs during CoV infection and suggest that DMVs can form passively in an atypical autophagy process. CoVs hijack autophagy initiation through NSPs and ORFs, which leads to non-nucleation phagophores and omegasomes to autophagy-derived DMVs.

Additionally, membrane rearrangement by hijacking ERAD machinery to form ER-derived DMVs independent from the autophagy process is another important routine for DMV production. Existing research also suggests that DMVs are not the only venues for CoV replication. Recent studies suggest that autophagosomes are also the sites where the virus can replicate, and autophagosomes that do not fuse with lysosomes can also rupture and release virus particles (Hui et al., 2021). This view is consistent with CoVs preventing the fusion of autophagosomes and lysosomes. Autophagosomes belong to a specific DMV. Therefore, virus particles are derived from DMV and autophagosomes. Here we also conclude that CoVs usurp host clearance *via* intercepting autophagy flux. CoVs usurp lysosome degradation by modulating lysosomal acidity or preventing autophagosome and lysosome fusion. SARS-CoV-2–infected patients should not suppress the autophagy–lysosome pathway to clinical treatment, which may not inhibit the replication of the virus. In a randomized clinical trial, although limited by early termination, there was no clinical benefit of hydroxychloroquine administered daily for 8 weeks as pre-exposure prophylaxis in hospital-based HCWs exposed to patients with COVID-19 (Abella et al., 2021). It has been proven that autophagic flux inhibitors such as hydroxychloroquine (HCQ) may not be effective drugs, but autophagic inducers may prove better alternatives (Abella et al., 2021; Devarajan and Vaseghi, 2021). Autophagy is the negative regulation of innate immunity, which could prevent the development of host cellular inflammation (Liu et al., 2019; Vera-Otaroola et al., 2020). The NSP3 PLP-TM domain is necessary to complex Beclin1 to STING1, which prevents the stimulation of IFN production (Zhang et al., 2021). However, CoVs escaping the innate immunity whether dependent on hijacking the autophagy–lysosome pathway is still unclear. Meanwhile, a molecular mechanism of the transportation and location of viral proteins is still mysterious.

Currently, various countries and regions have begun vaccination, from institutions such as Pfizer, Oxford University, and Sinovac Biotech. As of December 2021, more than 54% of the people have been vaccinated (Devarajan and Vaseghi, 2021). However, there are still no effective targeted drugs currently. Therefore, developing new drug targets and a new therapeutic strategy is urgent. Our review details which coronavirus NSPs and ORFs promote DMV formation, how CoVs evade autophagy–lysosome degradation and innate immunity, and how this may contribute to developing novel therapies and clinical drugs against COVID-19 in the future.

## Author Contributions

HWL and SL: conception and design and writing. HL and DL: revision of the manuscript. All authors read and approved the final manuscript.

## Conflict of Interest

The authors declare that the research was conducted in the absence of any commercial or financial relationships that could be construed as a potential conflict of interest.

## Publisher’s Note

All claims expressed in this article are solely those of the authors and do not necessarily represent those of their affiliated organizations, or those of the publisher, the editors and the reviewers. Any product that may be evaluated in this article, or claim that may be made by its manufacturer, is not guaranteed or endorsed by the publisher.

## Abbreviations

ATG: autophagy-related proteins
CQ: chloroquine
CoV: coronavirus
EDEM1: ER degradation enhancing alpha-mannosidase-like protein-1
DMV: double-membrane vesicles
ER: endoplasmic reticulum
IFN-I: type I interferon
IRF3: interferon regulatory factor-3
MERS: the Middle East respiratory syndrome
NSP: non-structural proteins
ORF: open-reading frame
SARS: severe acute respiratory syndrome
STING: stimulator of interferon gene
TLR: toll-like receptors
TBK1: TANK binding kinase 1
TRIF: TIR domain-containing adaptor inducing interferon- β .
